# Gene expression landscapes driving early life stages of the keystone seagrass *Posidonia oceanica*

**DOI:** 10.1007/s00299-026-03887-6

**Published:** 2026-06-26

**Authors:** Gianmarco Valenti, Alberto Sutera, Emanuela Dattolo, Francesco Cosenza, Francesco Carimi, Gabriele Procaccini, Francesco Mercati, Guglielmo Puccio, Roberto De Michele

**Affiliations:** 1 Institute of Biosciences and Bioresources (IBBR), CNR, Via Ugo La Malfa 153, 90146 Palermo, PA Italy; 2 Department of Biological, Chemical and Pharmaceutical Sciences and Technologies (STEBICEF), University of Palermo, Viale delle Scienze, 90128 Palermo, Italy; 3 Department of Integrative Marine Ecology, Stazione Zoologica Anton Dohrn, Villa Comunale, 80121 Naples, Italy

**Keywords:** Plant development, *Posidonia oceanica*, Seagrass, Seed, Seedling, Transcriptomics, WGCNA, Germination, Gene expression, Photosynthesis, Cell wall biogenesis, Carbohydrate metabolism

## Abstract

**Key message:**

Gene expression analyses reveal tissue-specific and temporally regulated networks driving *Posidonia oceanica* development, and identify key hub genes that coordinate root, leaf, and seed maturation.

**Abstract:**

Seagrasses are marine angiosperms that form extensive underwater meadows, providing habitat, stabilising sediments, storing carbon, and protecting coastlines. *Posidonia oceanica* is the endemic foundation seagrass of the Mediterranean Sea, yet its meadows are rapidly declining. Despite its ecological importance, the molecular basis of *P. oceanica* development is still poorly understood. Here, we analysed gene expression in roots, leaves, and seeds across four developmental stages, revealing strong tissue-specific patterns and temporally regulated expression dynamics. Leaves exhibited active regulation of photosynthesis-related processes, while roots were enriched in pathways related to carbohydrate metabolism and cell wall biogenesis, supporting primary root growth and anchoring. The seeds retained metabolic activity, with glycolytic enzymes indicating readiness for germination. Temporal analyses identified a major transcriptional shift, with distinct gene sets sequentially activated during early establishment and late maturation in tissues. Weighted Gene Co-expression Network Analysis (WGCNA) identified modules strongly associated with specific tissues and developmental transitions, highlighting key hub genes involved in photosynthesis, metabolism, cell wall remodelling, and protein synthesis. Together, these results reveal complex, temporally coordinated regulatory networks underlying *P. oceanica* development*.*

**Supplementary Information:**

The online version contains supplementary material available at 10.1007/s00299-026-03887-6.

## Introduction

Seagrass meadows are among the most valuable coastal ecosystems on Earth. They act as an erosion barrier that dampens swell, limits sediment movement on the seabed, and contributes to the sedimentary balance of beaches through the accumulation of biogenic sand (De Falco et al. 2017)*.*By providing food and shelter for fish and invertebrates, these meadows serve as vital nursery grounds for the fishing industry (Mtwana Nordlund et al. 2016). Furthermore, seagrass meadows act as carbon sinks, mitigating the global increase in carbon dioxide (Duarte and Krause-Jensen 2017). *Posidonia oceanica* (L.) Delile is the dominant seagrass in the Mediterranean Sea. However, anthropogenic pressure and climate change negatively impact the health and distribution of *P. oceanica* meadows, with documented regression reaching 34% over the past fifty years (Telesca et al. 2015). Therefore, targeted actions are essential to mitigate the drivers of this decline and to implement conservation practices to preserve key ecosystem functions.

*P. oceanica* reproduces both vegetatively, via lateral expansion of its rhizomes, and sexually, by releasing large buoyant fruits, which dehisce and expel a single seed. Unlike other species of seagrass, *P. oceanica* produces seeds that are not dormant and seedling development already begins within the fruit (Orth et al. 2000; Belzunce et al. 2005). Both lateral cuttings and beach-collected seeds have been used as propagation material for restoration efforts (Boudouresque et al. 2021). A seed-based strategy is cost-effective and preserves populations genetic diversity. However, it is hampered by a rapid decrease in seed viability among stranded seeds, unless they are protected by fruits or shielded from direct sunlight (Sutera et al. 2024). Early seed collection is essential; subsequently, seed viability can be preserved for several months under cold and low-light conditions, enabling the uncoupling of seedling development and transplantation from the initial time of collection (Sutera et al. 2025).

During early seedling development, the leaves and primary root emerge from the seed, which persists and nourishes the plantlet both with its starchy hypocotyl and through active photosynthesis up to the first year after seed release (Balestri et al. 2009; Celdran and Marin 2013; Celdran et al. 2015; Guerrero-Meseguer et al. 2018). Leaves rapidly elongate, and new pairs form to support photosynthesis (Sutera et al. 2025). At the same time, a primary root emerges, followed by secondary, adventitious roots anchoring the seedling to the substrate (Belzunce et al. 2008).

Recently, the genomes of *P. oceanica* and other seagrass species have been sequenced, revealing genomic adaptations to underwater marine life in those plant species that originally evolved on land (Ma et al. 2024a). However, to date, only a few studies have explored the patterns of gene expression in *P. oceanica* under physiologically normal and stress conditions, and all were performed on adult plants (Dattolo et al. 2013; Entrambasaguas et al. 2017; Procaccini et al. 2017; Marín-Guirao et al. 2019; Ruocco et al. 2021; Pazzaglia et al. 2022; Santillán-Sarmiento et al. 2023). Therefore, a global transcriptomic picture of the early stages of the *P. oceanica* lifecycle is still lacking.

This study addresses this critical gap in our current biological knowledge of *P. oceanica* by providing the first transcriptome analysis during seed germination and early seedling development: an analysis providing tissue-level resolution. Our results show that the seeds remained metabolically active, as indicated by the presence of glycolytic enzymes suggesting a state poised for germination. In parallel, roots predominantly expressed pathways involved in carbohydrate metabolism and cell wall formation, consistent with their role in early growth and establishment. Meanwhile, the leaves showed strong regulation of processes associated with photosynthetic activity. The identification of key genes and regulatory metabolic pathways that play a pivotal role in *P. oceanica* ontogeny could guide the selection of the best-performing seedlings as propagation material for meadow restoration projects.

## Materials and methods

### Seedling growth and experimental design

The stranded fruits were collected on the shore of Trabia, Sicily, Italy (37.994 N, 13.666 E) on May 19, 2022. The salinity in the area is 3.8% (w/v). At the time of collection, the air temperature was around 25°Cand the sky was clear. The seeds were removed from the fruit and placed under controlled conditions in an aquarium (40 L) with artificial seawater (3.8% salinity), biological filtration, controlled temperature (18 °C) and lighting regime (300 lx with a 14-h light/10-h dark photoperiod), with no substratum.

The first time point analysed (T0) consisted of three biological replicates of freshly collected seeds. The second time point(T1) consisted of three biological replicates for each tissue (leaf, root, and seed), which were sampled from seedlings one month after transfer to the aquarium. Two additional time points were analysed following the same procedure, but with seedlings selected at three months (T2) and six months (T3) after germination. For simplicity, throughout the manuscript, we refer to the seed remnant, attached to a developed seedling, as “seed”. Leaf and root length was measured from digital photographs using ImageJ software on five replicates. The total leaf length included older, brown leaves, whereas the total root length comprised all secondary branches of adventitious roots.

### RNA extraction

Leaf, root, and seed samples were collected at each time point and stored at -80 °C pending further processing. From each sample, aliquots of approximately 60–80 mg were ground into a fine powder under liquid nitrogen. The RNA from the leaf and root tissues was extracted from the powdered material with the Aurum Total RNA Mini Kit (BIO-RAD), following the manufacturer’s protocol for plant tissue. The seed samples contained a large amount of starch, which clogged the purification columns; therefore, the initial extraction phase was performed using the small-scale RNA Isolation procedure outlined in the PureLink Plant RNA Reagent (Thermofisher). Subsequently, the aqueous phase was processed using the Aurum Total RNA Mini Kit. Both the purity and the concentration of total RNA were assessed using a BioTek Synergy Plate Reader spectrophotometer. The integrity of the RNA was assessed by electrophoresis on a 1.0% (*w/v*) agarose gel. RNA concentrations, 260/280 ratios and RINs are provided in the Supplementary Table S1. Due to poor RNA quality, the samples obtained from the seed tissues in T2 were excluded from the analysis, resulting in a final dataset of 27 samples.

### RNA-seq analysis and differential expression analysis

The prepared samples were sequenced using an Illumina NovaSeq 6000 platform, following the Illumina protocol, to generate paired-end (PE) reads (Biodiversa, Italy). The quality of the raw sequencing data was assessed using FastQC (Wingett and Andrews 2018). The reads were then trimmed using TRIMMOMATIC (Bolger et al. 2014) with default parameters to obtain clean, high-quality reads with a median per-base sequence quality of 36 (Phred score) and no adapter sequences. Sequencing revealed a skewed GC content indicative of the presence of eubacteria. This was expected because the seedlings were grown under non-sterile conditions and because *P. oceanica* seeds already harbour a diverse community of endophytes (Crucitti et al. 2026). With this knowledge, mapping the reads against the reference genome of *P. oceanica* ensured the removal of non-*P. oceanica* sequences. The reads were assigned to the newly assembled genome of *P. oceanica* (Ma et al. 2024a, b) using the STAR tool (Dobin et al. 2013), with the default parameters. Finally, the mapped reads were assigned to genomic features using featureCounts (Liao et al. 2014), with the default parameters.

DESeq2 was used to perform differential expression analysis (Love et al. 2014). Raw counts were first normalised through DESeq2 size factor normalisation and transformed using the variance-stabilising transformation (VST) tool. Principal component analysis (PCA) was performed on the VST data. Tissue-specific differentially expressed genes (DEGs) were obtained through pairwise comparisons between tissues at each time point, using a false discovery rate (FDR) threshold of 0.001, a log2-fold change threshold of > 2.0 for upregulated, and < -2.0 for downregulated DEGs. The results of these multiple comparisons were visualised and analysed using an UpSet plot generated with the ComplexHeatmap R package (Gu 2022) to reveal shared DEGs between conditions.

In parallel, to capture genes with significant trends across different time points, a likelihood ratio test (LRT) was employed using a multifactor design. For this test, the entire model (~ Tissues + Time + Tissues:Time) was tested against a reduced model excluding the interaction term (~ Tissues + Time). Gene Ontology (GO) enrichment analysis was performed using the R package ClusterProfiler (Yu et al. 2012) with an adjusted p-value cut-off of 0.01 which was calculated using the Benjamini–Hochberg method. KEGG pathway enrichment analysis was performed using the R package clusterProfiler (Yu et al. 2012) with an adjusted alpha value cutoff of 0.05, calculated using the Benjamini–Hochberg correction method. Functional annotation of DEGs was carried out using the GhostKOALA web server (Kanehisa et al. 2016), which assigned KEGG Orthology (KO) identifiers by querying the KEGG GENES database. The results were visualised using the R package ggplot2(Wickham 2011).

Weighted gene co-expression network analysis (WGCNA) was applied to the VST data to identify co-expression modules. A soft thresholding power of 8 was selected to achieve scale-free topology (Supplementary Fig S1**)**. Modules were identified using dynamic tree cutting with a minimum size of 30 genes. For each module, the associations were calculated as the Pearson correlation coefficient between the eigengene (the principal component of the module expression profile) and a binary matrix representing all experimental conditions. Network visualisation was performed in Cytoscape and the hub genes were defined as the top 5% of the most connected genes within each module.

### Validation of gene expression

For the expression analysis of target genes, total RNA extracted from leaf, root, and seed tissues collected at time point T3 was used. Three independent biological replicates were analysed for each tissue. A total of six target genes showing overexpression in specific organs based on RNA-seq analysis data were selected for expression profiling across all tissues: *Posoc06g08810* and *Posoc05g15670* for leaves; *Posoc05g19340* and *Posoc01g30140* for roots; and *Posoc04g01410* and *Posoc10g10780* for seeds. Reverse transcription into cDNA was performed using the QuantiTect reverse transcription kit (Qiagen) according to the manufacturer’s protocol. For each biological replicate, a target amount of 80 ng of total RNA was used per single reverse transcription reaction. To ensure a sufficient template for subsequent quantitative real-time PCR (qPCR) analyses, two independent reaction rounds were performed for each sample and then merged to a final volume of 40 µl. RT-qPCR was performed with FastStartTM SYBR® Green Master Mix (Roche) using a CFX Connect Real-Time PCR Detection System (Bio-Rad Laboratories) in a total reaction volume of 25 µl. For each biological replicate, three technical replicates were included, each containing 1.4 µL of cDNA template. Negative controls, performed in triplicate, lacked a cDNA template.

Amplification was carried out with the following parameters: initial denaturation at 95 °C for 10 min, 40 cycles of denaturation at 95 °C for 15 s, annealing at 57 °C for 45 s and extension at 72 °C for 30 s. A final melting curve analysis was performed from 57 °C to 95 °C with a heating rate of 0.5 °C every 5 s. The reference genes *Posoc10g04890* (*EIF4a*) and *Posoc05g07530* (*GAPDH*) were amplified using the same protocol, except for the annealing temperature (53 °C). Primers were designed to span the regions between two exons, whenever possible, to avoid residual genomic DNA amplification. All primers used for RT-qPCR are listed in Supplementary Table S2.

### Statistical analysis

The number of leaves and roots for each developmental stage is presented as the mean along with the associated standard deviation (n = 5 independent seeds/seedlings). Pairwise differences were statistically tested using the *t*-test. The lengths of leaves, roots, and primary roots among developmental stages were compared using the Kruskal–Wallis test, followed by pairwise Mann–Whitney tests (alpha = 0.05).

For transcriptome analyses, *n* = 3 independent seeds/seedlings were used. Statistical processing of transcriptome data, including the Wald test for pairwise comparisons, the LRT for temporal trends, and the WGCNA, has been described in detail in Sect. 2.3.

For qPCR, we considered the mean C_t_ of three technical replicates per sample. The ΔC_t_ was calculated by subtracting the C_t_ value of the reference genes (calculated as the geometric mean of the C_t_ values of *EIF4a* and *GADPH*) from the C_t_ value of the target gene. Means and standard deviations were calculated among the three biological replicates. Pairwise differences were tested by a one-way ANOVA, followed by Tukey’s honestly significant difference (HSD) post hoc test (alpha = 0.05). Relative expression levels among tissues were presented as 2^−ΔCt^.

## Results

### Seedling development

**Fig. 1:**
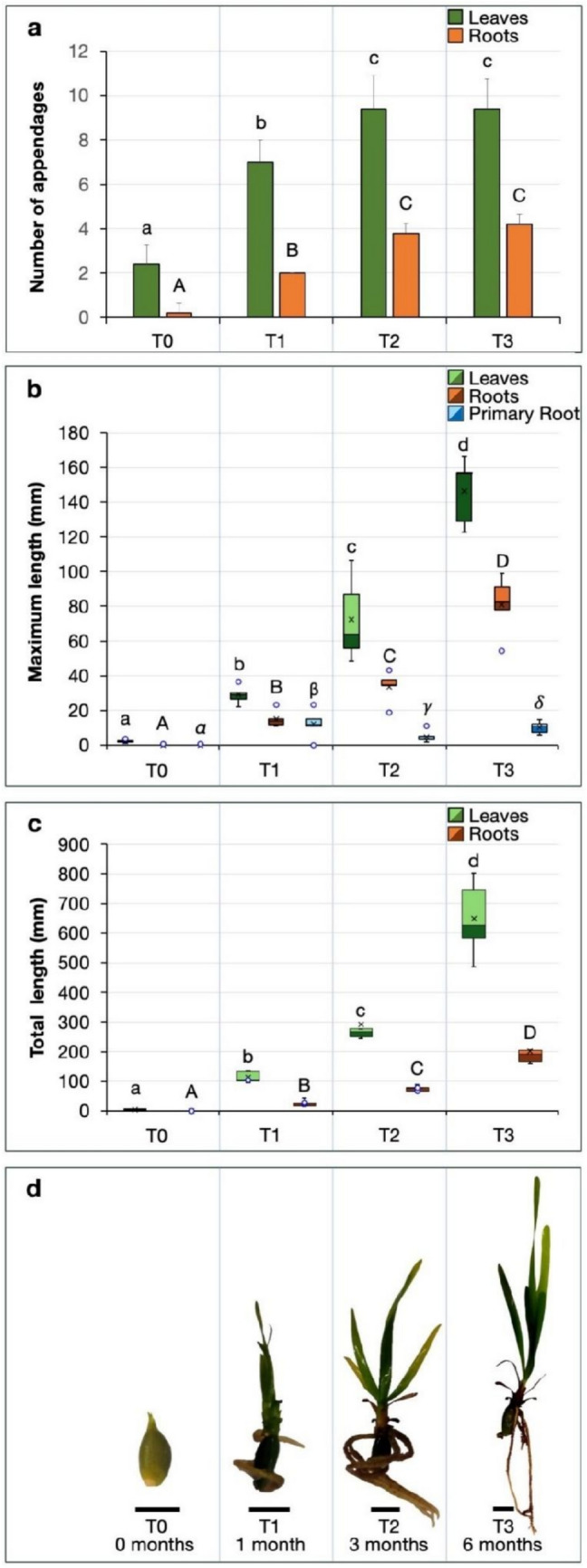
Development of seeds. **A** Number of leaves and roots; means and standard deviations are presented. **B** Maximum leaf, root, and primary root lengths; means are presented as crosses, and medians as lines dividing boxes. **C** Total leaf and root lengths; means are presented as crosses, and medians as lines dividing boxes. **D** Representative images of seedlings at each stage of development. Bar = 1.0 cm. Different letters indicate significant differences between time points within each tissue

The seeds germinated rapidly in the aquarium, with pairs of leaf primordia having formed in the seed apex prior to germination in many individuals (Supplementary Fig S2). Furthermore, the leaf primordia expanded rapidly during growth. After one month, seven leaves had formed, and an additional pair appeared soon after (Fig. 1A). The leaves elongated, reaching approximately 15 cm after 6 months (Fig. 1B). A single primary root appeared on the opposite side of the seed, its growth was variable, and ceased after the first month in all seeds (Fig. 1B, Supplementary Fig S2). Secondary, adventitious roots developed later at the rhizome junction, increasing in number and length during the cultivation period (Figs. 1A, B). At six months, the branching of secondary roots was also evident (Fig. 1D, Supplementary Fig S2). The trends in total leaf and root length paralleled those of maximum single leaf and root lengths (Fig. 1B, C).

### Transcriptional profiling of tissues of *P. oceanica*

A total of 1.1 billion PE reads were mapped to the reference genome, averaging 42 million reads per sample. On average, 18,478 ± 294 (SD) unique genes were detected, representing 79% of the total gene set.

**Fig. 2:**
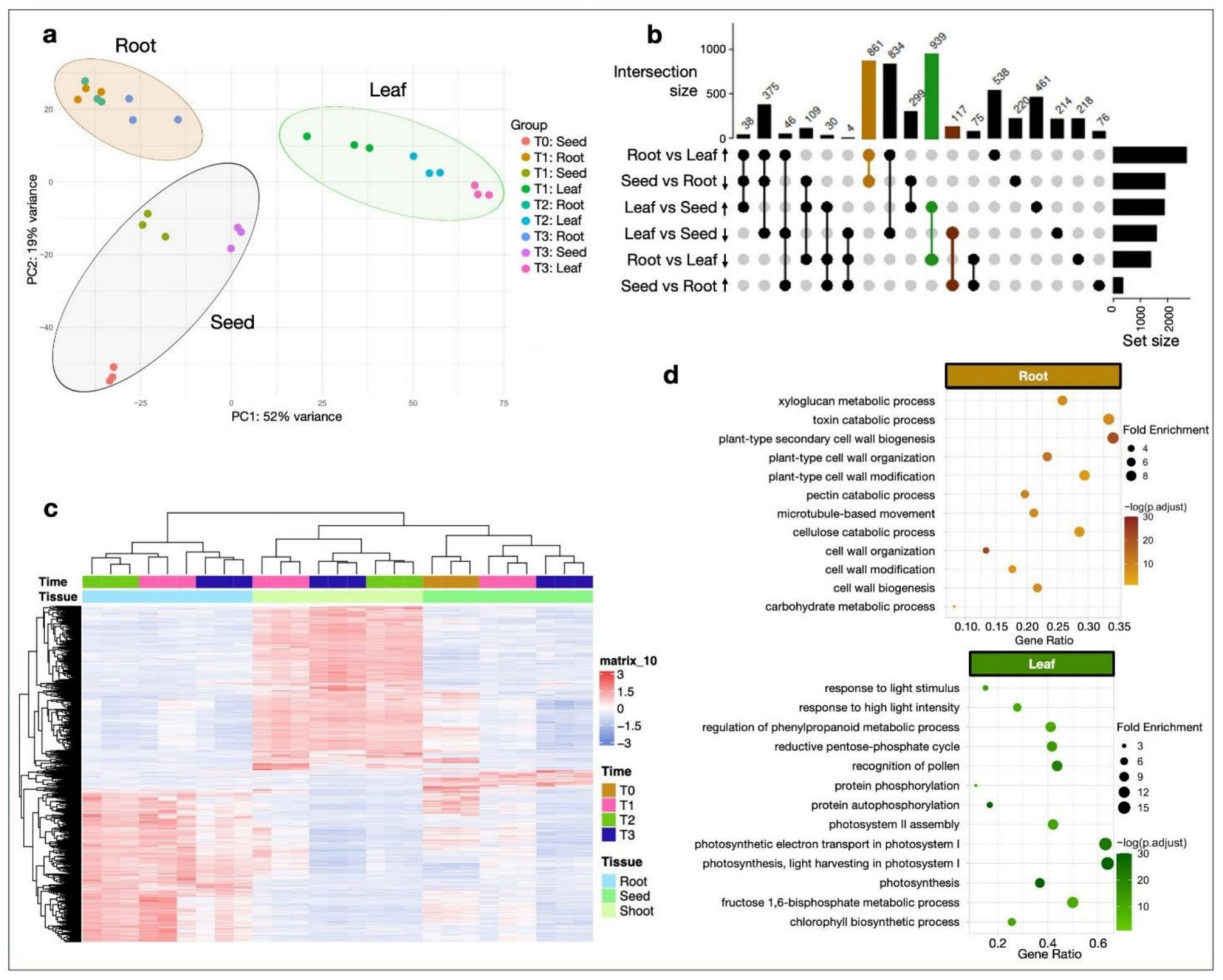
Tissue and temporal distribution of gene expression**. A** PCA performed on three tissues (root, leaf, seed) at different time points (0, 1, 3, and 6 months after germination) of *P. oceanica* seedlings, *n* = 3 biological replicates. **B** UpSet plot displaying the intersection patterns of DEGs identified in pairwise contrasts. Tissue-specific DEGs are colour-coded: leaf (green), root (light brown), and seed (dark brown). Bars represent intersection sizes, with connected dots indicating contributing comparison sets, and numbers on each bar representing the number of DEGs. **C** Heatmap showing the expression patterns of tissue-specific genes. **D** Dot plot showing GO enrichment results for biological processes in leaf- and root-specific genes

PCA of the 27 *P. oceanica* samples revealed a clear separation of biological replicates according to tissue type. Specifically, samples grouped into three distinct clusters corresponding to root, leaf, and seed samples (Fig. 2A). The first two principal components (PC) explained most of the variance, with PC1 and PC2 accounting for 53% and 19% of the variance, respectively.

Pairwise contrasts between tissues revealed extensive transcriptional differences during distinct stages of development with a large number of DEGs identified. The identified DEGs were further filtered to differentiate between up- and down-regulated genes (log2 fold change >  ± 2). Overall, 4009 DEGs (2660 upregulated and 1349 downregulated) were detected in root versus leaf samples, 2101 (304 upregulated and 1797 downregulated) in the seed versus root samples, and 3397 (1794 upregulated and 1503 downregulated) in the leaf versus seed samples (Supplementary Table S3).

To define tissue-specific gene sets, the analysis has focused on DEGs that were specifically induced in a single tissue in all pairwise comparisons. By cross-referencing the upregulated DEGs across all comparisons (e.g., Leaf vs. Seed up and Root vs Leaf down), 939 unique DEGs specific to leaves, 861 for roots, and 117 to seeds were identified (Fig. 2B). This comparative approach allowed the filtering of those genes specifically activated during the early development of a single tissue relative to the rest of the plant tissues (Fig. 2B; Supplementary Table S4). A heatmap of these tissue-specific DEGs displayed their expression profiles across the four developmental stages (T0–T3). Hierarchical grouping revealed consistent clustering of samples by tissue type and developmental stage (Fig. 2C).

To gain insight into the biological functions of these DEGs, GO enrichment analysis was performed. Leaf-specific DEGs were predominantly enriched in photosynthesis-related processes, photorespiration, light stimulus, and metabolic regulation. Root-specific DEGs were enriched in carbohydrate metabolism and cell wall-related processes, including the xyloglucan metabolic processes and cell wall organisation (Fig. 2D). Seed-specific DEGs showed a more restricted enrichment, with only one significant term detected in biological processes, specifically the polysaccharide catabolic process (Supplementary Table S4). To further characterise the functional roles of the identified DEGs, a KEGG pathway enrichment analysis was performed to investigate the biological pathways in which these genes are involved. Seed-specific DEGs did not produce any significantly enriched pathways. In contrast, leaf-specific DEGs showed enrichment in pathways consistent with the GO results, including photosynthesis, carbon fixation by the Calvin cycle, and phenylpropanoid biosynthesis. Root-specific DEGs were enriched in pathways related to phenylpropanoid biosynthesis and starch and sucrose metabolism. (Supplementary Fig. S3).

### Time-dependent modulation of gene expression during tissue development

A LRT was applied to identify genes showing tissue- and time-specific transcriptional dynamics, revealing 4642 DEGs (*p*_*adj*_ < 0.001). Hierarchical clustering grouped these genes into nine clusters: Cluster 1 (382 DEGs), Cluster 2 (1080), Cluster 3 (365), Cluster 4 (832), Cluster 5 (199), Cluster 6 (210), Cluster 7 (588), Cluster 8 (840), and Cluster 9 (146) (Supplementary Table S5).

**Fig. 3:**
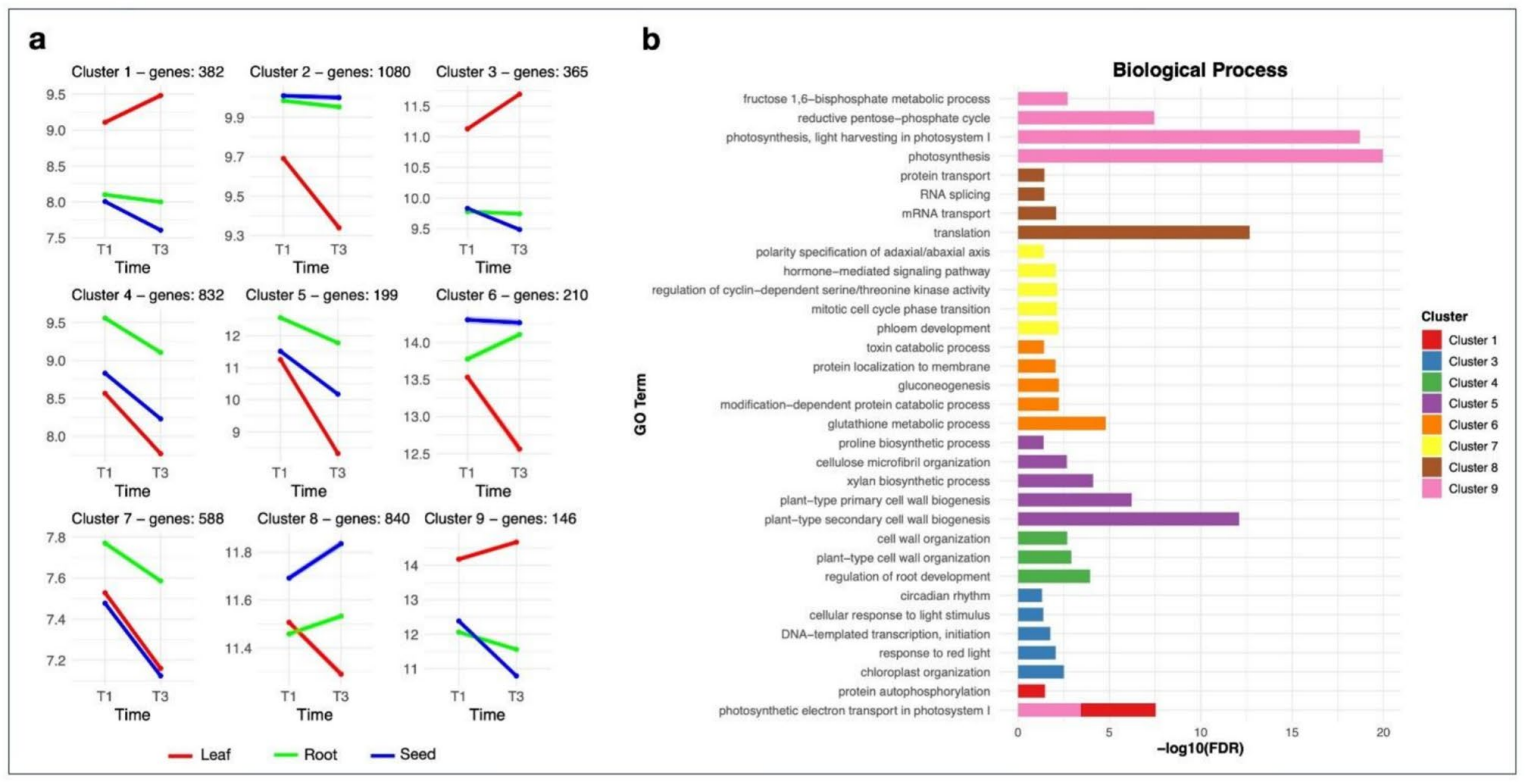
Expression patterns and associated GO terms. **A** Dot plot showing temporal trends of clusters obtained by LRT analysis for the interaction between Time and Tissue. **B** Bar plot showing the 5 most enriched GO terms (biological processes, BP) of the identified clusters

Analysis of expression patterns (Fig. 3A) highlighted tissue- and time-dependent trends. Clusters 1, 3, and 9 showed progressive upregulation in leaves and down-regulated expression in roots and seeds. The expression of Cluster 2 genes remained stable in roots and seeds, but was downregulated in leaves. Clusters 4, 5, and 7 exhibited a general downregulation during the assessment period, with moderate increases in roots, but a pronounced decrease in expression levels in leaves and seeds.

The functional enrichment of these groups ($\mathtt{P}$ adj ≤ 0.01) revealed biologically relevant patterns (Fig. 3B), with distinct temporal regulation across different tissues. In leaf samples, clusters with up-regulated expression over time were enriched in photosynthesis-related terms, including “photosynthetic electron transport in photosystem I” and “cellular response to light stimulus”. Progressive downregulation over time characterised the clusters associated with structural development and growth. This trend was specifically observed in Clusters 4 and 5, which are involved in cell wall organisation and biogenesis, as well as in Cluster 7, linked to hormonal regulation of cell division.

In contrast, other functional groups exhibited tissue-specific regulation. For example, Cluster 6 was enriched in metabolic and stress response processes, such as gluconeogenesis and glutathione metabolism, showing an induction in roots and seeds while being downregulated in leaves. A similar tissue-dependent pattern was observed for Cluster 8, where RNA splicing and mRNA transport were prominent in seeds and roots, but declined in leaf tissues.

### Analysis of co-expression networks

**Fig. 4:**
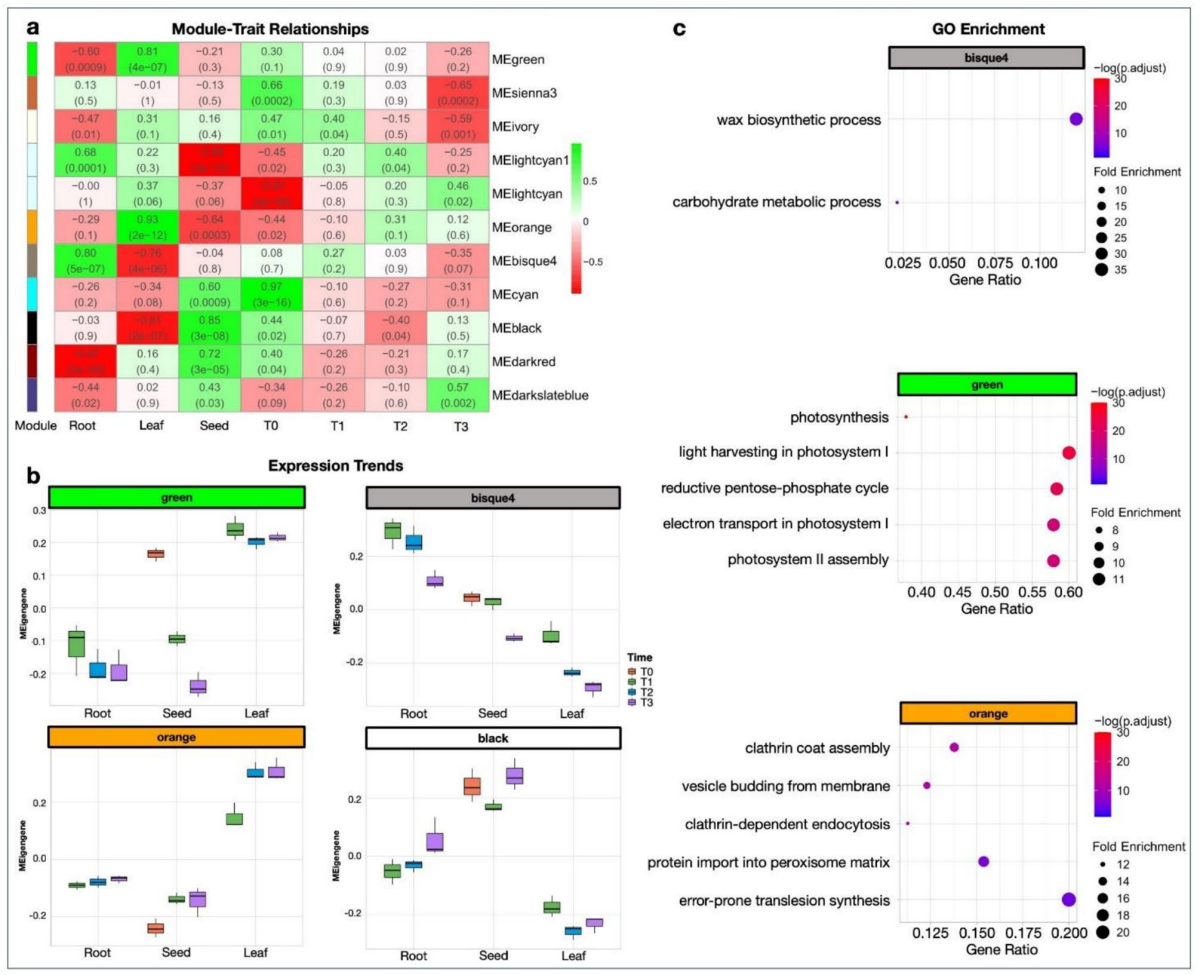
WGCNA of *P. oceanica* samples. **A** Module-trait relationships revealed by the Pearson correlation coefficient. The leftmost colour column indicates different co-expression modules. The numbers in the figure indicate the correlation between the modules and traits, and the numbers in parentheses are the correlation *p*-values. **B** Module eigengene temporal trend across WGCNA modules in which tissue-specific genes are grouped (“green”, “orange”, “black”, and “bisque4”). **C** Biological Process GO enrichment analysis of the corresponding modules showing the functional distribution within the "bisque4", "green", and "orange" modules

To uncover co-expression gene networks underlying *P. oceanica* development, we performed a WGCNA. All transcriptomic profiles, characterised by a total of 18,299 genes, were grouped by clustering analysis, and a sample correlation matrix was calculated. The analysis led to the identification of a total of 11 co-expression modules after the merging phase, each represented by a distinctive colour, as shown in Fig. 4. These ranged from 91 (“ivory” module) to 5189 genes (“bisque4” module), with the second largest module being “orange” (4531 genes).

The correlation analysis between module eigengenes and experimental conditions (treated as a binary matrix) comprising time (T0, T1, T2, T3) and tissue (root, leaf, seed) allowed the selection of specific modules correlated with time points and tissues (Fig. 4A). Four modules showed significant correlations with specific tissues (|*r*|> 0.6): “bisque4” and “lightcyan1” with roots, “orange” and “green” with leaves, and four smaller modules (“dark slate blue”, “dark red”, “black”, and “cyan”) with seeds.

Modules showing significant correlations with developmental time were also identified. The distribution of module activity showed specific temporal patterns across the experimental timeline. The T0 time point was characterised by the significant association of several modules, specifically “sienna3”, “ivory”, “cyan”, “black”, and “darkred”. The “ivory” module was the only one to maintain a significant correlation through the T1 time point. In later stages, the transcriptional profile shifted towards stage-specific modules: “lightcyan1” showed a peak correlation at T2, while at T3 the expression was dominated by the coordinated activity of the “darkslateblue” and “lightcyan1” modules.

To identify modules with tissue-specific expression patterns, DEGs from pairwise contrasts were mapped onto WGCNA modules. Almost 96% of root-specific DEGs were assigned to the “bisque4” module (823 DEGs), which was enriched in functions involved in “carbohydrate metabolic process” and “wax biosynthetic process”. Leaf-specific DEGs (930) were distributed in the “green” and “orange” modules (~ 33% and 55%, respectively). The “green” module was enriched in GO terms related to photosynthetic activity, such as “photosynthesis”, “light harvesting in photosystem I” and “photosystem II assembly”, while the “orange” module was associated with detoxification mechanisms, including “protein import into the peroxisome matrix” and “vesicle-mediated endocytosis/exocytosis processes”, such as “clathrin-dependent endocytosis”. Seed-specific DEGs (101) were mapped primarily to the “black” module (58 DEGs) which did not show significant enrichments.

### Co-expression modules involved in tissue differentiation and identification of key molecular regulators

**Fig. 5:**
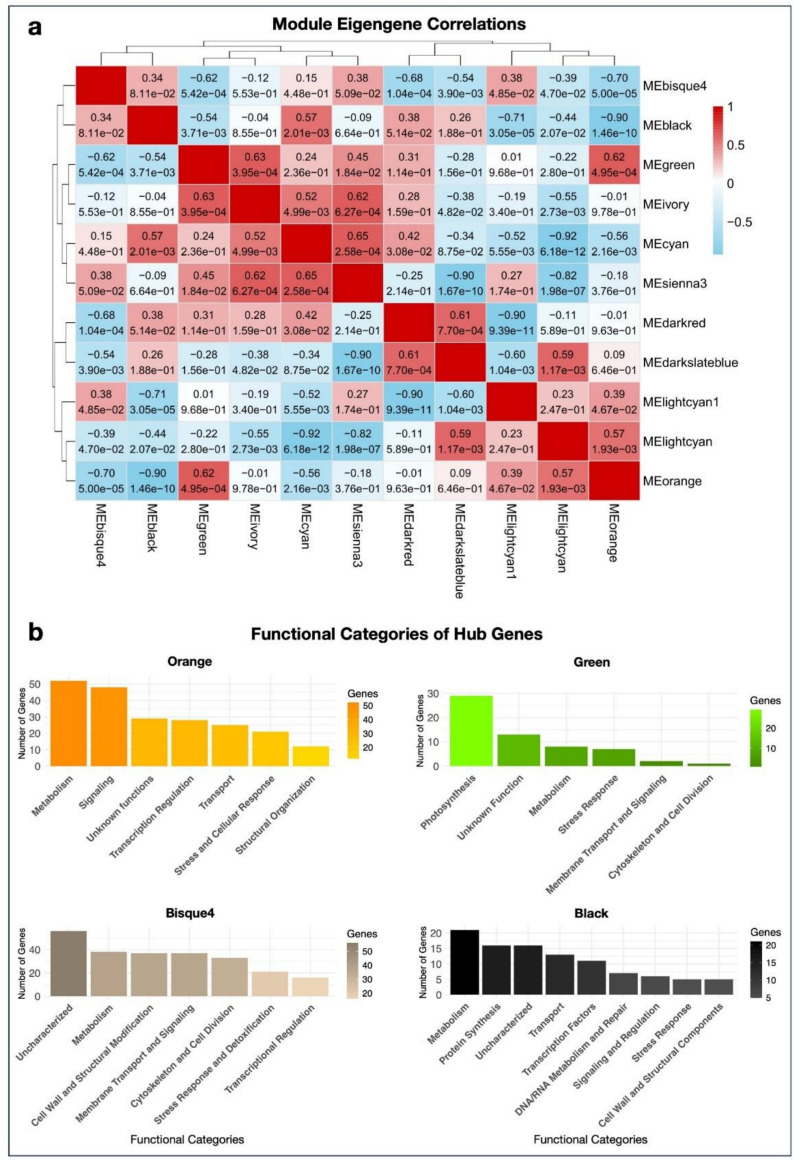
Correlation and functional analyses of hub genes. **A** Heatmap of module-module eigengene correlations. The dendrogram and colour scale represent the hierarchical relationship between co-expression network analysis modules, where red indicates positive and blue indicates negative eigengene correlation. **B** Bar plot showing the functional categories of hub genes identified in the “black”, “bisque4”, “green”, and “orange” co-expression modules of *P. oceanica* via WGCNA. The X-axis reports the functional categories of the identified hub genes, while the Y-axis indicates the corresponding number of genes for each category

The co-expression modules were further characterised through correlation analysis of the module eigengenes, network analysis, and functional analysis of the hub genes (Fig. 5; Supplementary Fig S4, Supplementary Table S6). The eigengene correlation analysis revealed the global transcriptional architecture of *P. oceanica* development, with modules showing similar expression profiles clustering together (Fig. 5A). Hierarchical clustering of module eigengenes revealed a sharp architectural divergence between tissue-specific transcriptional programmes. Specifically, modules associated with the seeds and roots (“black” and “bisque4”) clustered independently from leaf-associated modules (“green” and “orange”). Although the latter two showed a high correlation coefficient (*r* = 0.62), their separation into distinct subclades highlights their specialized biological functions: photosynthesis for the “green” module and broader metabolic signaling for the “orange” module.

The seed development-associated “black” module was dominated by transcriptional control (11 genes), including regulators, such as *ZAT9*, a zinc finger transcription factor involved in embryo development through gibberellin and ethylene responses (Joseph et al. 2014). Protein turnover specialists (16 genes) maintained proteostasis, while DNA/RNA processors (7 genes) ensured genetic stability. Metabolic hubs (21 genes) fuelled growth through glycolysis and pentose phosphate pathways, supported by transporters (13 genes) for reserve accumulation and stress protectants (5 genes) to protect developing embryos.

Analysis of the root development-associated “bisque4” module highlighted specialized networks for marine adaptation, characterised by structural genes (37 genes) for cell wall remodeling, metabolism regulators (38 genes), and signaling hubs (37 genes) coordinating nutrient uptake. Cytoskeletal organisers (33 genes) enabled cell division, while stress responders (21 genes) mitigated marine oxidative stress and transcriptional regulators (16 genes) guided root patterning.

Modules associated with leaf development showed distinct specialisations. The “green” module concentrated on chloroplast function (29 genes) and metabolic support (8 genes), with additional roles in stress resilience. In contrast, the “orange” module exhibited broader regulatory roles, primarily in metabolic diversification (49 genes), signaling networks (48 genes), and transcriptional control for processes, such as iron homeostasis and morphogenesis.

### Validation of gene expression patterns

**Fig. 6:**
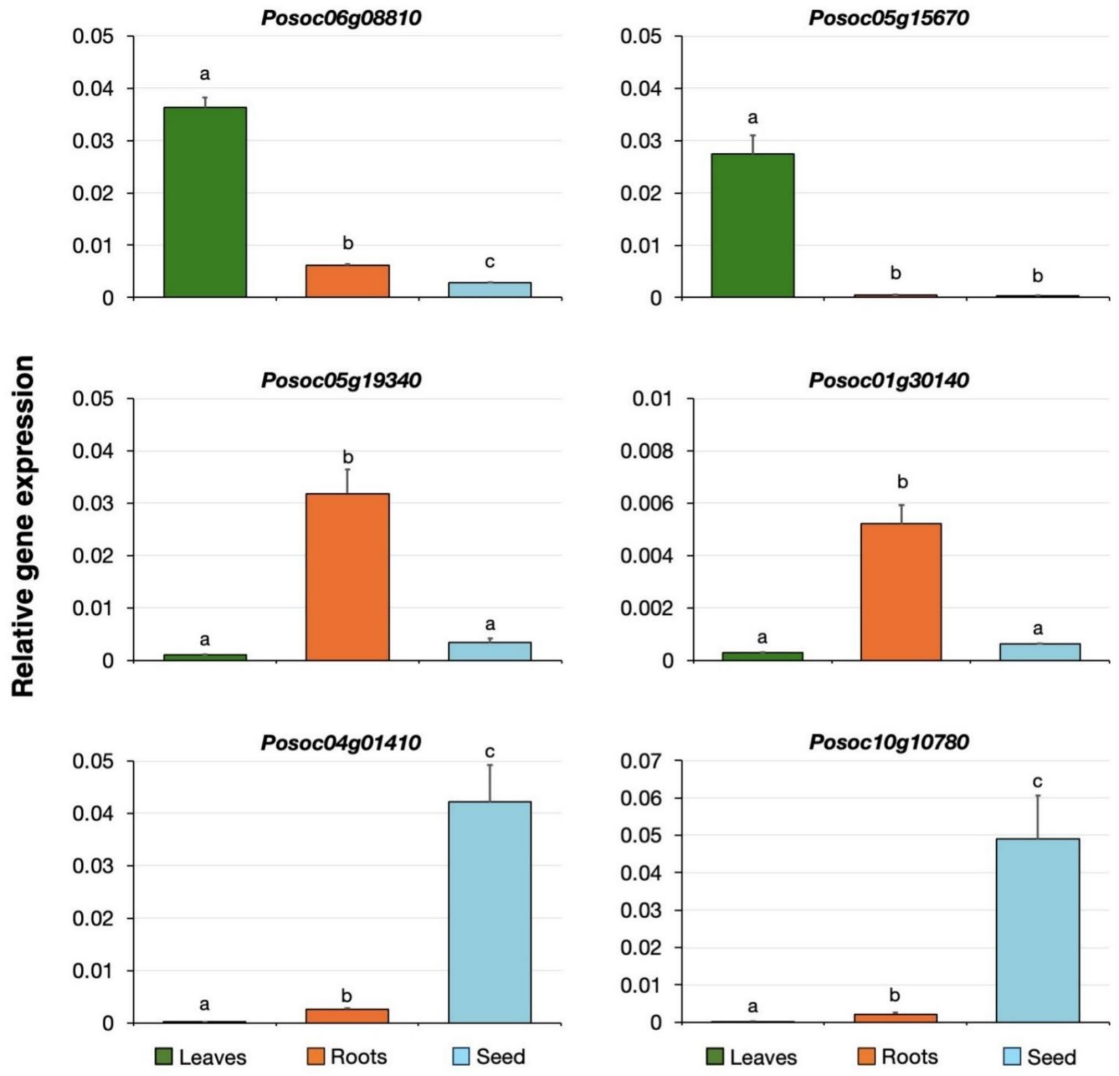
Relative gene expression levels of target genes**.** Expression profiles of *Posoc06g08810*, *Posoc05g15670*, *Posoc05g19340*, *Posoc01g30140*, *Posoc04g01410*, and *Posoc10g10780* quantified by RT-qPCR in leaves, roots, and seeds of six-month-old seedlings (T3). Different letters indicate significant differences among tissues

To ensure the reliability of the transcriptomic analysis, we validated the expression patterns of a subset of six genes by qPCR. These genes were selected from the differentially expressed hub genes identified through transcriptomic analysis and included: two genes overexpressed in leaves (*Posoc06g08810*, encoding a carotenoid $\phi$-ring synthase; and *Posoc05g15670,* encoding a UDP-glycosyltransferase; both belonging to the “orange” module); two genes overexpressed in roots (*Posoc05g19340*, encoding a MYB-related protein; and *Posoc01g30140*, encoding a zinc finger protein; both belonging to the “bisque4” module); and two genes overexpressed in seeds (*Posoc04g01410*, encoding a UDP-glycosyltransferase; and *Posoc10g10780*, encoding anSRC2 protein; both belonging to the “black” module). The qPCR results confirmed that all six genes showed high expression levels in their respective tissues compared to the other tissues (Fig. 6). Amplifications resulted in single amplicons of the expected sizes.

## Discussion

Our study aimed at uncovering the molecular processes underlying *P. oceanica* seedling development across three key tissues (roots, leaves, and seeds) and four developmental stages. To achieve this, we combined differential gene expression analysis, temporal dynamics modelling via a likelihood ratio test (LRT), and weighted gene co-expression network analysis (WGCNA). This integrated transcriptomic approach revealed clear tissue-specific patterns in gene expression, with distinct transcriptomic modules dedicated to photosynthesis in leaves, structural and metabolic adaptation in roots, and energy mobilisation in seeds. These findings demonstrate how specialised functions shape different tissues alongside dynamic temporal regulation throughout developmental stages.

Principal Component Analysis (PCA) demonstrated that tissue type is the primary determinant of transcriptional variation, accounting for the majority of the observed variance (72%). The clear separation of samples unequivocally identifies tissue identity as the primary driver of seedling transcriptome. This finding is consistent with transcriptomic studies in model plants, such as Arabidopsis and crop species, such as rice and chickpea, where tissue origin consistently emerges as the strongest driver of gene expression patterns (Schmid et al. 2005; Brady et al. 2007; Kudapa et al. 2018).

In particular, the PCA revealed a clear pattern in which the T0 time point marks the starting phase of seedling differentiation into leaf and root tissues. This likely reflects the major transcriptomic reprogramming characteristic of the seed-to-seedling transition during early development (Smolikova and Medvedev 2022). This is consistent with the morphoanatomical structure of *P. oceanica* seeds, which possess a well-developed plumule and radicle primordia at maturity (Belzunce et al. 2005), thereby occupying an intermediate position between fully developed leaf and root tissues.

Time-tissue interactions further clarified these functional shifts. Clusters 1, 3, and 9 were enriched in photosystem I/II genes, highlighting the contribution of leaf tissues to photosynthesis. This is consistent with the molecular mechanisms underlying the production of photosynthates in response to light availability, as previously described in *P. oceanica* (Procaccini et al. 2017). In contrast, Clusters 4, 5, and 7, which are associated with cell growth and hormonal regulation, reflected root developmental processes, corroborating brassinosteroid-mediated salinity responses (Rodríguez-Rojas et al. 2024).

### Sequential activation of transcriptional programs during development

The enriched functions of DEGs identified by the LRT comprise clusters of GO terms highly linked to the functional specialisation of individual tissues during plant development. Clusters 1, 3, and 9, which showed progressive upregulation in leaf tissues, were enriched in terms related to photosynthesis, including electron transport in photosystem I and cellular response to light stimulus. This result confirmed the central role of leaves during the early development of *P. oceanica* and the importance of regulating photosynthetic function during development. This is consistent with findings in other angiosperms, such as pearl millet (Wu et al. 2021), where a significant enrichment of genes linked to photosynthetic activity was observed.

In contrast, Clusters 4, 5, and 7, which were associated with cell wall development, showed a decreasing trend of expression across all tissues, with higher expression levels in roots. This suggests that the regulation of cell wall production is important for the development of all tissues, but particularly for the development of the root system. This trend is analogous to that observed in other plants, such as maize (Stelpflug et al. 2016; Somssich et al. 2016). This pattern likely corresponds to the end of the initial phase of cell elongation, a hormonally regulated process supported by Cluster 7, which underscores the importance of hormonal regulation during root system development, consistent with findings during root elongation (Ma et al. 2024b).

Furthermore, the activation in seeds (Cluster 6) of key metabolic pathways, such as gluconeogenesis and glutathione metabolic, processes is biologically coherent with the germination phase. Gluconeogenesis is crucial for converting seed lipid reserves into glucose, which is necessary for energy provision during early growth (Walker et al. 2021), while increased glutathione metabolism could be related to the regulation of seed germination processes (Koramutla et al. 2021).

### Metabolic and signaling responses govern leaf development

We observed a significant enrichment of genes associated with photosynthetic processes, with 930 genes directly involved in pathways, such as “photosynthesis”, “photosystem assembly”, and “photosynthetic electron transport”. This result confirmed the central role of leaves as the primary photosynthetic organ in *P. oceanica*. Additionally, terms related to pigment biogenesis, such as “chlorophyll biosynthetic process” and “carotenoid biosynthetic process”, were enriched, indicating strong activity in chloroplast assembly and the synthesis of photosynthetic pigments, which are essential for early development and the construction of the photosynthetic apparatus. Previous studies have already emphasised the importance of these pathways in leaves during light acclimation, as shown by Dattolo et al. (2017) while Ruocco et al. (2019) highlighted the central role of carotenoids in photoprotection mechanisms under thermal stress conditions.

The results showed that photosynthetic tissue (i.e., leaves) actively regulates processes, such as the fructose-6-phosphate metabolic process, gluconeogenesis, and the reductive pentose-phosphate cycle, to support growth of the entire plant. Similar regulatory patterns have been observed in other angiosperms. For example, studies on tomato have shown how fructose-6-phosphate metabolism and gluconeogenesis are central to stem development (Cai et al. 2016), while in Arabidopsis, the reductive pentose-phosphate cycle is crucial for the metabolic balance of leaves (Borghi et al. 2019). Furthermore, the enrichment of genes associated with “protein phosphorylation” and “protein autophosphorylation” indicates a central regulatory role of kinases and signaling pathways, which has already been observed in response to light acclimatisation in adult leaves of *P. oceanica* (Dattolo et al. 2017). Furthermore, the involvement of the “phenylpropanoid metabolic process” is relevant because of its role in lignification, structural stability, and antioxidant defence of tissues, as described by Xing et al. (2024). Finally, some defensive response terms, such as “response to chitin”, highlight the activation of immune pathways related to the recognition of fungal elicitors, in agreement with the observations of Ruocco et al. (2019).

Further analysis of leaf-associated co-expression modules strengthened these observations. The strong positive correlation observed in the module-eigengene correlation analysis suggested that these modules are part of a synchronised, leaf-specific transcriptional branch. This coordination likely ensures that photosynthetic output (“green” module) is tightly coupled with the signaling networks and nutrient homeostasis (“orange” module) required for optimal leaf performance in a marine environment.

The “green” module, specifically linked to leaf tissue, was largely composed of genes involved in photosynthesis (particularly components of photosystems I and II) and other key metabolic processes, in line with previously described depth-related modulation of photosynthetic pathways in *P. oceanica* (Procaccini et al. 2017). Key players included ferredoxin, chlorophyll a/b binding proteins, and RuBisCO, which stabilise photosystem II and optimise light responses. Ribosomal genes, e.g. encoding for 50S ribosomal protein L1, further underscored the importance of chloroplast maintenance, aligning with previous findings reported by Tourasse et al. (2013). Notably, the absence of transcription factors in this module suggests a streamlined network dedicated to photosynthetic efficiency.

The “orange” module, also associated with leaf tissue, seemed to complement the “green” module by focusing on leaf transcriptional regulation, exhibiting broader regulatory roles and encompassing transcriptional control, signaling, and metabolic diversification. Genes encoding for enzymes, such as invertase and cystathionine gamma-synthase 1, mirrored those active in *Zostera marina* during vegetative growth (Zhao et al. 2025), while stress-responsive transcription factors (such as AP2 / ERF, WRKY, and bHLH) and signaling genes (eg, LRR-RK) reflected adaptations critical for development, growth, and survival in the marine environment, as seen in *Z. marina* (Zhao et al. 2025).

### Transcriptomic regulation underpins root structural development

Our study identified 861 specific DEGs involved in key functions underlying *P. oceanica* root system development, such as polysaccharide metabolism, cell wall biogenesis, structural remodelling, and environmental response. Upregulation of genes related to functions, such as “xyloglucan metabolic process”, “pectin catabolic process,” and “cellulose catabolic process”, is fundamental to producing components that drive the expansion and elongation of the root system. These remodelling processes were consistent with the dynamic nature of the cell wall described for seagrasses, which exhibit specific adaptations in the composition of cellulose, hemicellulose, and pectin (Pfeifer and Classen 2020). Such processes are sustained by energy generated through carbohydrate metabolism, which exhibits pronounced seasonal variability and metabolic plasticity in *P. oceanica* (Ismael et al. 2023).

The high expression of genes involved in cell wall biogenesis and cell wall organisation suggests that *P. oceanica* roots undergo intense deposition of cellulose, lignin, and other structural components, confirming what is known about *P. oceanica* cell wall composition (Pfeifer and Classen 2020). Finally, consistent with a developing and elongating tissue, such as the root system, functions related to the cytoskeleton, such as “microtubule-based movement”, highlight the role of the cytoskeleton in regulating cell elongation and orienting the deposition of cellulose microfibrils. These processes provide rigidity and mechanical strength, essential characteristics to ensure plant stability within the sediment and facilitate nutrient transport. Similar structural properties have been documented in terrestrial plants, where cortical microtubules guide cellulose synthase complexes, ultimately shaping cell wall organisation and mechanical strength (Lei et al. 2014).

The “bisque4” module, highly upregulated in roots, further linked wax biosynthesis and carbohydrate metabolism to structural reinforcement, in agreement with findings on lignin-mediated hydrophobicity in *P. oceanica* roots (del Río et al. 2022). Hub genes additionally emphasised sterol biosynthesis pathways (eg, ATP-citrate synthase, δ(24)-sterol reductase), consistent with light-responsive metabolism described in *Z. marina* (Kumar et al. 2017). The presence of MYB transcription factors, which resemble those active in salt-stressed *Z. marina* (Zhao et al. 2025), suggests that conserved regulatory mechanisms control the process of root specialisation across different seagrass species. Interestingly, the eigengene correlation heatmap revealed a distinct transcriptional distance between the root-associated “bisque4” module and the leaf-associated modules. This separation highlights the high degree of functional specialisation in roots, which operate under a regulatory regime distinct from autotrophic tissues.

These pathways are fundamental to modulating the elasticity and plasticity of the cell wall during root growth. Interestingly, *P. oceanica* seedling roots have been shown to adhere firmly to the substrate, withstanding forces up to 2.4 N and effectively resisting the environmental forces of strong storms (Zenone et al. 2020). This adhesion is ascribed to the mechanical interlocking of roots due to the plasticity of root hairs, which grow along the microroughness of the substrate and adapt to the microtopography of the surface, rather than through the secretion of adhesion compounds. This scenario is consistent with our findings, which highlighted intense cell wall remodelling activity and microfibril deposition. Taken together, these results reflect the high functional specialisation of the root tissue, operating not only for anchorage and nutrient absorption, but also as a metabolically active organ capable of adapting to the often extreme conditions of marine sediment.

### Transcriptomic activity sustains metabolic and regulatory responses in seeds

This study enabled the identification of key mechanisms that guide early germination and transcriptomic activity in seeds during development. The seed of *P. oceanica* is an active tissue that maintains its functional capacity into advanced stages of development. Our results indicate that the breakdown and utilisation of starch and sugars provide energy and carbon sources during the early stages of germination. This was evidenced by the analysis of 101 seed-specific DEGs identified via pairwise comparisons, which were enriched in “carbohydrate metabolism”, a process crucial for early development. These results agree with observations in the seagrass *Z. marina* (Zhang et al. 2023), where carbohydrate-related pathways were markedly upregulated during early germination.

The “black” module, although not functionally significantly enriched, harbored genes pivotal for germination, such as carbohydrate-metabolising enzymes (including enolase and pyruvate kinase). These findings align with studies of *Z. marina* (Zhu et al. 2023) that highlighted the key role of carbohydrates as energy reserves during early growth. In particular, our transcriptome data revealed sustained metabolic activity in *P. oceanica* seeds even after germination, suggesting continued resource mobilisation, a trait critical for seedling establishment in marine environments. This is confirmed by previous analyses of *P. oceanica* seed content, which reported a decrease in carbon, nitrogen, and phosphorus in seeds during the first months after germination due to nutrient reallocation to leaves and roots (Balestri et al. 2009).

Interestingly, we did not observe modulation of genes involved in photosynthesis in seed tissue. This stood in contrast to multiple physiological observations indicating that seeds act as functionally active photosynthetic organs during the development of *P. oceanica* seedlings (Celdran and Marin 2013; Celdran et al. 2015; Guerrero-Meseguer et al. 2018). It is conceivable that photosynthesis is restricted to the outermost cell layer of the seed, therefore, the upregulation of photosynthetic machinery remains unnoticed because of dilution by the bulk of the inner seed, which is more active in reserve mobilisation.

Hierarchical proximity between seed and root modules highlighted a shared molecular signature that distinguished these tissues from the photosynthetic organs. The correlation between the “black” and “bisque4” modules pointed to their combined role in the breakdown of external nutrients and the reorganisation of internal cellular components, which sustains *P. oceanica* development before the photosynthetic apparatus reaches full maturity.

Furthermore, the presence of transcription factors, such as the NAC domain-containing protein 87 (NAC87) and MYB73, analogous to those governing dormancy in seashore paspalum (Wu et al. 2024) and the production of fatty acids in Arabidopsis (Yang et al. 2025), respectively, further implies dual roles in stress response and developmental control beyond the germination phase. These elements further highlight the complexity and fine-tuned control that govern *P. oceanica* seed germination, positioning the seed as a metabolically versatile organ capable of supporting seedling establishment in a challenging marine environment.

### Conclusions

This study provided a comprehensive transcriptomic analysis of seedling ontogeny in the ecologically important seagrass *P. oceanica*. By examining gene expression across roots, leaves, and seeds, we identified distinct tissue-specific signatures and regulatory modules that underpin early development. Our analysis revealed temporally regulated gene clusters governing early and late developmental stages, as well as functionally specialised clusters with dynamic expression profiles over time, illustrating the complex regulatory networks that guide *P. oceanica* development under controlled conditions.

Collectively, these results enhance our understanding of the molecular basis of *P. oceanica* development, providing insights into its ecological roles in coastal ecosystems, including sediment stabilisation, carbon storage, and biodiversity support. The tissue-specific and temporally regulated gene networks identified here provide a solid foundation for future functional studies, particularly to elucidate how *P. oceanica* adapts to the marine environment. Future research should focus on validating these hub genes through functional genomics and exploring their expression dynamics under natural environmental conditions to further inform conservation strategies for this critical Mediterranean species.

Furthermore, the genes identified in this study may serve as valuable molecular biomarkers for assessing the fitness and development programs of seedlings used in restoration initiatives. Because these early life stages represent a bottleneck for *P. oceanica* viability*,* and meadow restoration remains a costly endeavour, management practices would benefit significantly from the employment of the best-performing propagation material.

## Supplementary Information

Below is the link to the electronic supplementary material.

Supplementary file1 (DOCX 18 KB)

Supplementary file2 (DOCX 17 KB)

Supplementary Table S3 Up-regulated and down-regulated genes in pairwise comparisons of leaves, roots, and seeds Supplementary file3 (XLSX 303 KB)

Supplementary Table S4 Differentially expressed genes in leaves, roots, and seeds Supplementary file4 (XLSX 101 KB)

Supplementary Table S5 Differently expressed genes and their functions expression arranged in clusters of expression Supplementary file5 (XLSX 330 KB)

Supplementary Table S6 Hubgenes, organised in expression modules supplementary file6 (XLSX 43 KB)

Supplementary file7 (DOCX 602 KB)

## Data Availability

The RNA-Seq data underlying this article are available in NCBI-SRA, and can be accessed with the reference number PRJNA1434360.
