# Supplementary material for: Gene expression landscapes driving early life stages of the keystone seagrass Posidonia oceanica
Source: Plant Cell Rep. 2026 Jun 26;45(7):208. doi: 10.1007/s00299-026-03887-6 (PMC13309366; doi:10.1007/s00299-026-03887-6)
Supplement: Supplementary file 1 — Supplementary file1 (DOCX 18 KB) [file 299_2026_3887_MOESM1_ESM.docx]

Gene Expression Landscapes Driving Early Life Stages of the Keystone Seagrass *Posidonia oceanica*

*Plant Cell Reports*

Gianmarco Valenti, Alberto Sutera, Emanuela Dattolo, Francesco Cosenza, Francesco Carimi, Gabriele Procaccini, Francesco Mercati, Guglielmo Puccio, Roberto De Michele

Institute of Biosciences and Bioresources (IBBR), CNR., Via Ugo La Malfa 153, 90146 Palermo, PA, Italy

roberto.demichele@cnr.it

| **Time point** | **Tissue** | **Replicate** | **Concentration (ng/µL)** | **260/280** | **RIN** |
| --- | --- | --- | --- | --- | --- |
| T0 | seed | A | 29.8 | 1.95 | 6.9 |
| T0 | seed | B | 62.4 | 1.86 | 7.7 |
| T0 | seed | C | 25.2 | 1.99 | 7.1 |
| T1 | leaves | A | 197 | 2.2 | 8.2 |
| T1 | leaves | B | 94 | 2.15 | 8.2 |
| T1 | leaves | C | 145 | 2.15 | 8.4 |
| T1 | roots | A | 46.4 | 2.03 | 9.7 |
| T1 | roots | B | 39 | 2.09 | 9.3 |
| T1 | roots | C | 25.2 | 2.04 | 9.5 |
| T1 | seed | A | 53 | 1.58 | 8.2 |
| T1 | seed | B | 104 | 1.66 | 6.7 |
| T1 | seed | C | 12.5 | 1.81 | 6.7 |
| T2 | leaves | A | 26.8 | 2.05 | 7.2 |
| T2 | leaves | B | 112 | 2.17 | 6.9 |
| T2 | leaves | C | 26 | 2.11 | 7.1 |
| T2 | roots | A | 22.6 | 1.84 | 8.9 |
| T2 | roots | B | 37.6 | 2.06 | 9.7 |
| T2 | roots | C | 21.4 | 1.98 | 8.9 |
| T2 | seed | A | 103 | 1.53 | 6.5 |
| T2 | seed | B | 7.4 | 1.74 | 2.6 |
| T2 | seed | C | 12.3 | 1.61 | 3.1 |
| T3 | leaves | A | 70.4 | 2.45 | 6.2 |
| T3 | leaves | B | 99.4 | 2.31 | 6.2 |
| T3 | leaves | C | 84.4 | 2.35 | 6.5 |
| T3 | roots | A | 27.6 | 1.77 | 8.9 |
| T3 | roots | B | 41.4 | 2.0 | 9.2 |
| T3 | roots | C | 21.2 | 1.93 | 8.3 |
| T3 | seed | A | 29.8 | 1.49 | 6.2 |
| T3 | seed | B | 4.6 | 1.27 | 7.3 |
| T3 | seed | C | 10 | 1.62 | 7 |

**Supplementary Table S1. Quantity and quality of exracted RNAs.**
