# Supplementary material for: Gene expression landscapes driving early life stages of the keystone seagrass Posidonia oceanica
Source: Plant Cell Rep. 2026 Jun 26;45(7):208. doi: 10.1007/s00299-026-03887-6 (PMC13309366; doi:10.1007/s00299-026-03887-6)
Supplement: Supplementary file 7 — Supplementary file7 (DOCX 602 KB) [file 299_2026_3887_MOESM7_ESM.docx]

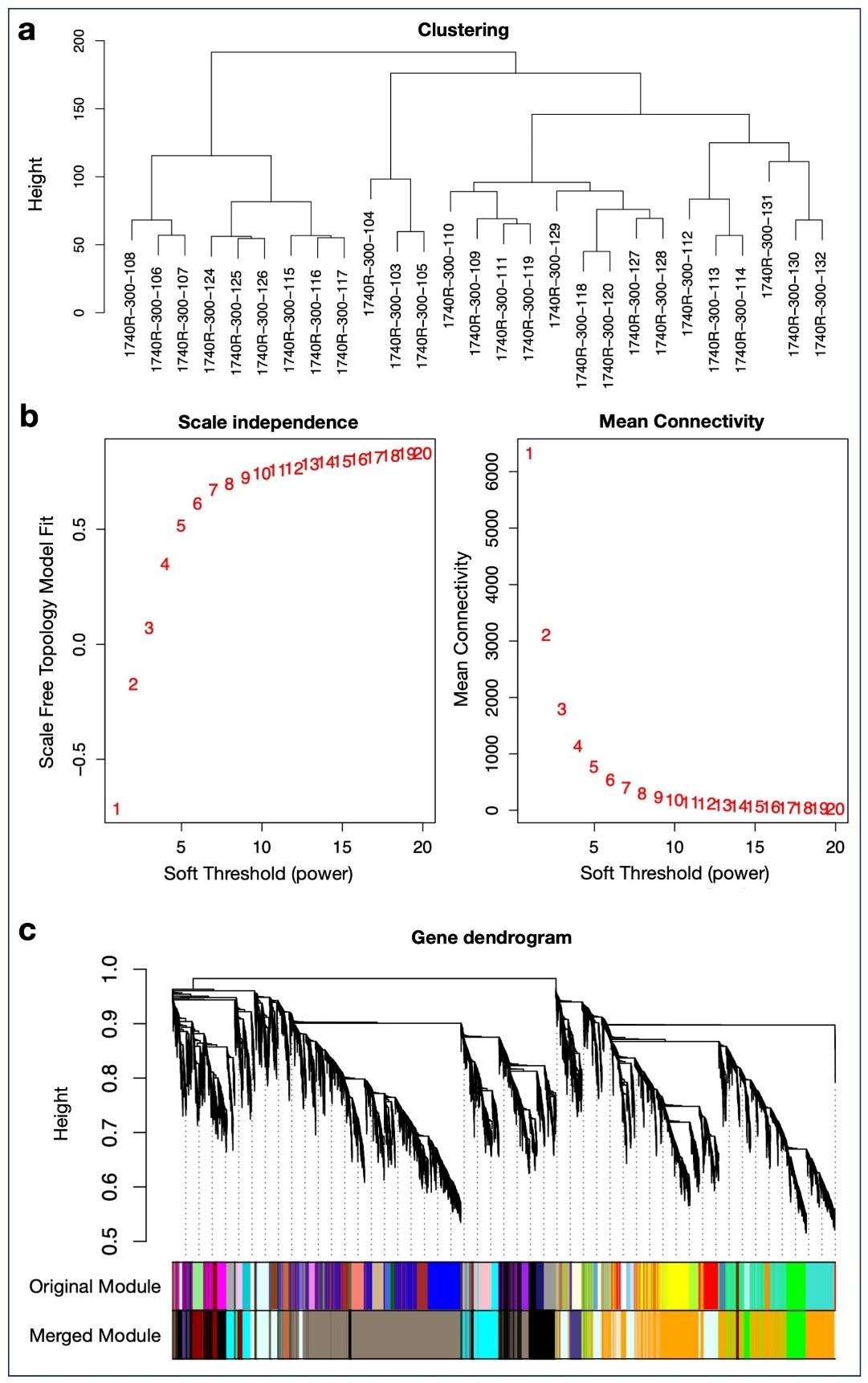


**Supplementary Fig. S1** (A) Sample clustering based on global gene expression profiles used to assess sample similarity and identify potential outliers prior to network construction. (B) Soft-tresholding power selection for WGCNA. The left panel shows the scale-free topology fit index across increasing powers, while the right panel shows the corresponding mean connectivity values. (C) Gene dendrogram generated from hierarchical clustering of the topological overlap matrix, with modules assigned through dynamic tree cutting and subsequently merged based on eigengene similarity. Colours denote distinct WGCNA modules.


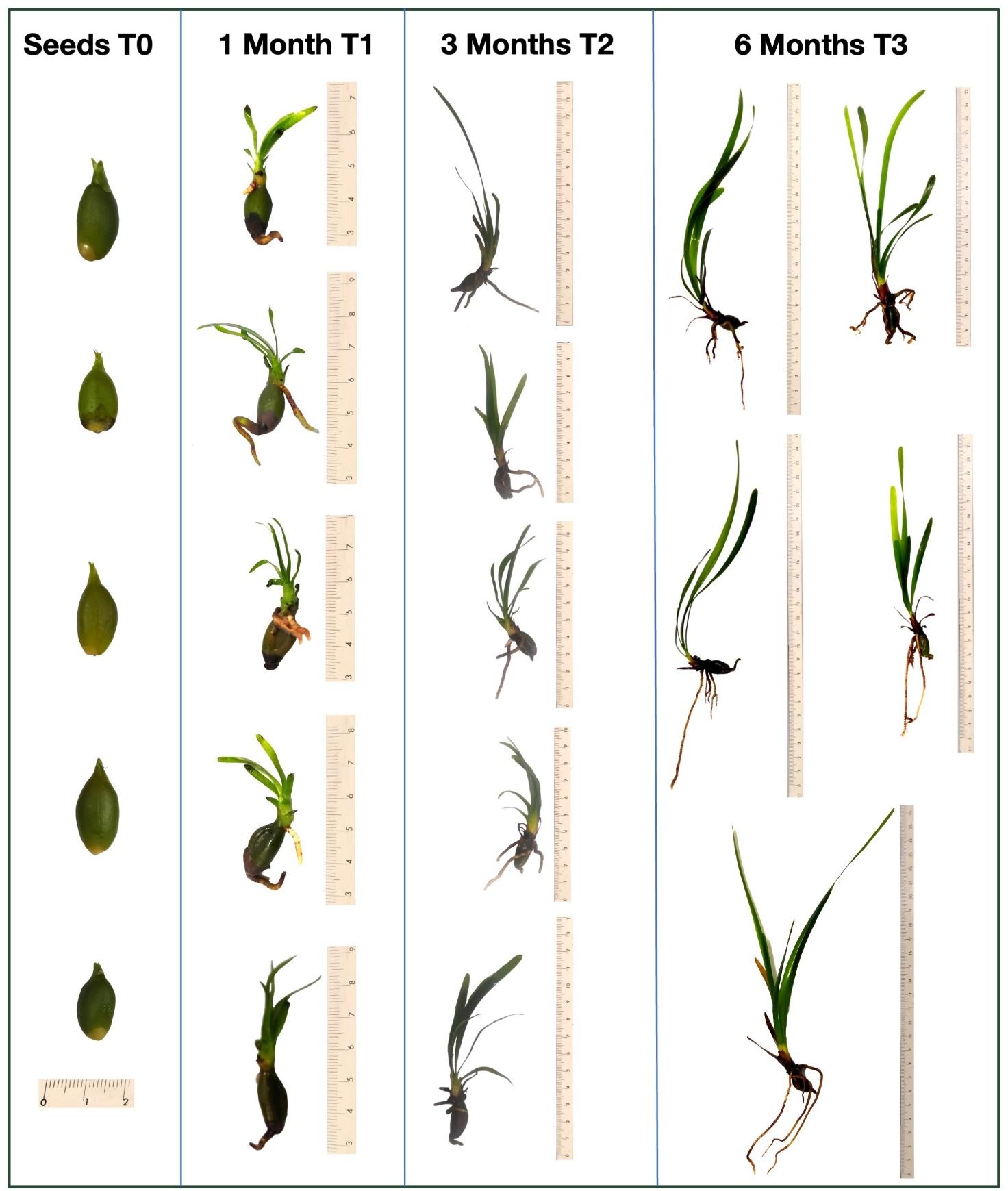


**Supplementary Fig. S2** ***P. oceanica* seedlings analysed in this study.** The three upper seedlings of each developmental stage were further processed for transcriptomic analyses.


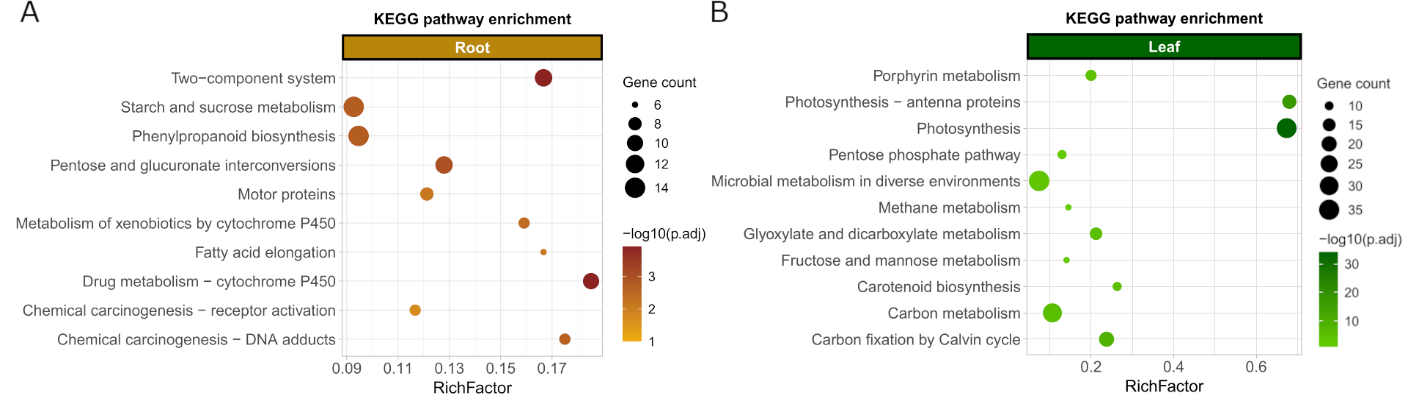


**Supplementary Fig. S3.** **KEGG pathway enrichment analysis of differentially expressed genes in the root and leaf tissue of *Posidonia oceanica*.** The size represents the number of genes enriched in each pathway, while the intensity of the colour indicates the statistical significance expressed as log10 (p.adjust). Tissue-specific DEGs are colour-coded. A**:** root (light brown); B**:** leaf (green).


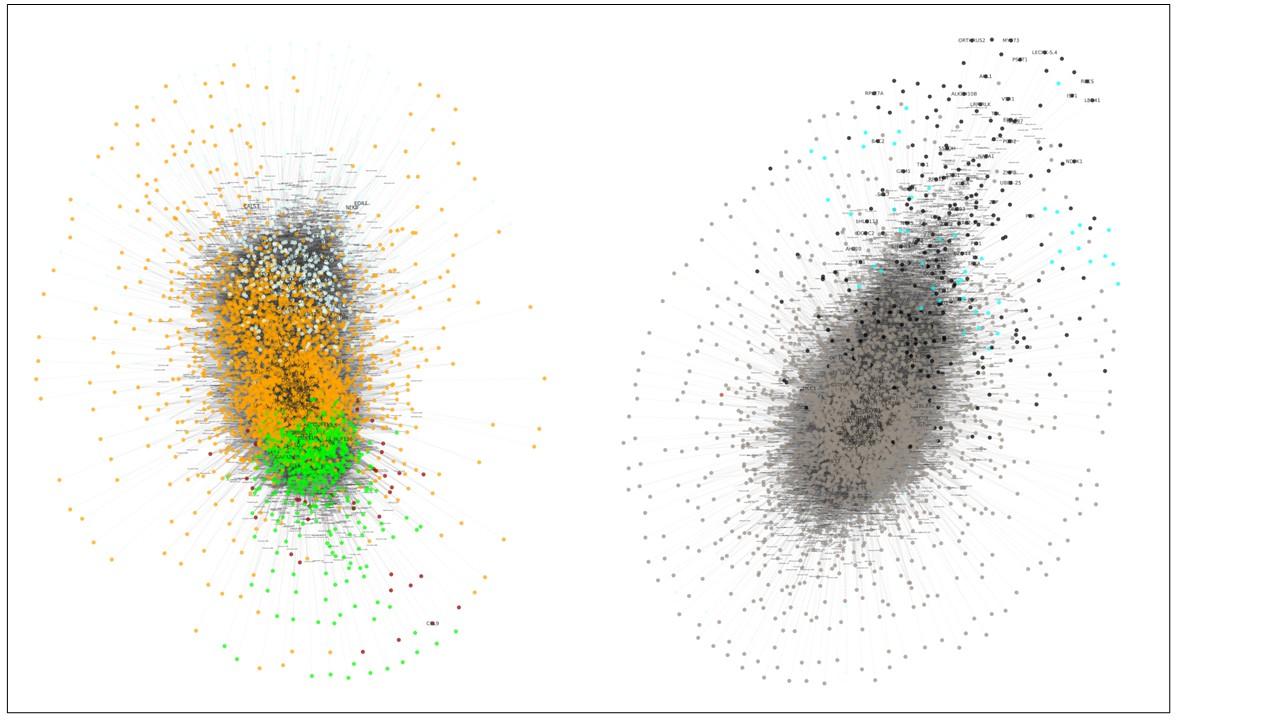


**Supplementary Fig. S4** Network visualization of WGCNA modules from developmental analysis.
